# Physiological and Anatomical Differences and Differentially Expressed Genes Reveal Yellow Leaf Coloration in Shumard Oak

**DOI:** 10.3390/plants9020169

**Published:** 2020-02-01

**Authors:** Xiaoyun Dong, Libin Huang, Qingsheng Chen, Yunzhou Lv, Hainan Sun, Zhenhai Liang

**Affiliations:** Jiangsu Academy of Forestry, Nanjing 211153, China; hongguo417@163.com (X.D.); nerring@163.com (Q.C.); lky209210@outlook.com (Y.L.); sunhainan1989@hotmail.com (H.S.); hongguo613@163.com (Z.L.)

**Keywords:** shumard oak, leaf color, chlorophyll, carotenoids

## Abstract

Shumard oak (*Quercus shumardii* Buckley) is a traditional foliage plant, but little is known about its regulatory mechanism of yellow leaf coloration. Here, the yellow leaf variety of *Q. shumardii* named ‘Zhongshan Hongjincai’ (identified as ‘ZH’ throughout this work) and a green leaf variety named ‘Shumard oak No. 23’ (identified as ‘SO’ throughout this work) were compared. ‘ZH’ had lower chlorophyll content and higher carotenoid content; photosynthetic characteristics and chlorophyll fluorescence parameters were also lower. Moreover, the mesophyll cells of ‘ZH’ showed reduced number of chloroplasts and some structural damage. In addition, transcriptomic analysis identified 39,962 differentially expressed genes, and their expression levels were randomly verified. Expressions of chlorophyll biosynthesis-related glumly-tRNA reductase gene and Mg-chelatase gene were decreased, while pheophorbide a oxygenase gene associated with chlorophyll degradation was up-regulated in ‘ZH’. Simultaneously, carotenoid isomerase gene, z-carotene desaturase gene, violaxanthin de-epoxidase gene and zeaxanthin epoxidase gene involved in carotenoid biosynthesis were up-regulated in ‘ZH’. These gene expression changes were accompanied by decreased chlorophyll content and enhanced carotenoid accumulation in ‘ZH’. Consequently, changes in the ratio of carotenoids to chlorophyll could be driving the yellow leaf coloration in *Q. shumardii*.

## 1. Introduction

Leaf color, the same as flower color, is very important for ornamental plants. Especially in autumn, when the leaf color of the autumn leaf tree changes, which attracts people’s attention. In order to increase the ornamental effect of a garden, colorful leaf tree species are widely used in gardens, such as red maple (*Acer rubrum* L.) of red leaves [1], gingko (*Ginkgo biloba* L.) of yellow leaves [2], etc. The direct cause of leaf discoloration is the change in contents of chlorophyll, carotenoids, anthocyanins and other pigmented substances, which are affected by both inheritance and environmental factors, and are regulated by leaf cell microstructure and metabolism [3,4,5]. At present, most of the research focuses on red leaf discoloration. For example, Chen et al. [1] found that the accumulation of cyanidin and reduction of chlorophyll and carotenoids produced the redness of red maple leaves. Gu et al. [6] found that the anthocyanin content of the leaves of *Prunus cerasifera* increased under light induction, and the leaves gradually turned purple. In contrast, although leaf yellowing is a common phenomenon, related research is still rare [2]. However, the yellowing of leaves is getting in the focus of current research. For example, Li et al. [2] reported that lower chlorophyll and higher carotenoid content (especially lutein) are the main factors for yellowing of gingko leaves, and Chang et al. [7] found that the ratio of chlorophyll a/b and carotenoid to chlorophyll increased in the leaves of yellow leaf mutant (*yl1*) of tree peony.

In addition to the fact that the pigment content in the leaves directly affects leaf color, the anatomical structure of the leaves and related genes also play important roles in leaf coloration. For example, the chloroplast of the crape myrtle (*Lagerstroemia indica*) yellow leaf mutant did not develop well [8], and the number of chloroplasts contained in the green leaves of the New Year’s Day orchid (*Cymbidium sinense*) was much larger than that of yellow leaf [9]. The related genes mainly control the biosynthesis and degradation of pigments by regulating the synthesis of related enzymes, thereby controlling leaf coloration. Because chlorophyll biosynthesis involves a series of enzymatic steps, any step that blocks the process results in low chlorophyll content, resulting in leaf green defects [10,11]. The chlorophyll synthesis process involves more than 15 enzymes and more than 20 genes [11]. Important genes in the synthesis process, such as glumly-tRNA reductase gene (*HEMA*), Mg-chelatase gene (*CHLD*/*CHLH*), chlorophyllide an oxygenase gene (*CAO*), etc., all decreased in expression during the leaf color transformation [8,12,13]. Chlorophyll degradation is also one of the main causes of leaf discoloration. The chlorophyll-b reductase genes (*NOL*/*NYC*) play a key role in chlorophyll degradation. For example, *NYC1* and *NOL* in rice form a complex that functions as a chlorophyll b reductase to regulate chlorophyll degradation [14]). The important genes related to plant carotenoid biosynthesis are phytoene synthase gene (*PSY*) [15], z-carotene desaturase gene (*ZDS*) [16], β-cyclase gene (*LCYB*) [17], ε-cyclase gene (*LCYE*) [18,19] and so on. Gao et al. [20] found that the activity of phytoene desaturase (PDS) in grapefruit callus was inhibited, resulting in a significant reduction in total carotenoid content. In addition, processes resulting in leaf discoloration also affect photosynthesis in plants. Zhang et al. [21] found that net photosynthesis rate (*Pn*) and chlorophyll fluorescence parameters of the yellow leaf areas of *Aucuba japonica variegata* were significantly lower than those in the green areas. Aluru et al. [22] also detected elevated *Pn* in the green leaf portion of the *Arabidopsis thaliana* variegation mutant.

Shumard oak (*Quercus shumardii* Buckley) is a common garden landscaping tree, esteemed for its leaves turning red in autumn, which has a good ornamental value. Under optimum growth conditions, leaves of *Q. shumardii* are generally green. We obtained a yellow leaf variety of *Q. shumardii* named ‘Zhongshan Hongjincai’ (identified as ‘ZH’ throughout this work), and a green leaf variety named ‘Shumard oak No. 23’ (identified as ‘SO’ throughout this work). The mechanism of yellow leaf coloration of ‘ZH’ is still unknown. In this study, their color indices were firstly measured, and then, the related pigment content, photosynthetic characteristics and chlorophyll fluorescence parameters were measured; subsequently, the anatomical structures of leaf mesophyll cells were observed; finally, comparative transcriptomic analysis was performed, and their related metabolic pathways and the expression patterns of key genes were investigated.

## 2. Results

### 2.1. Color Indices

**Figure 1:**
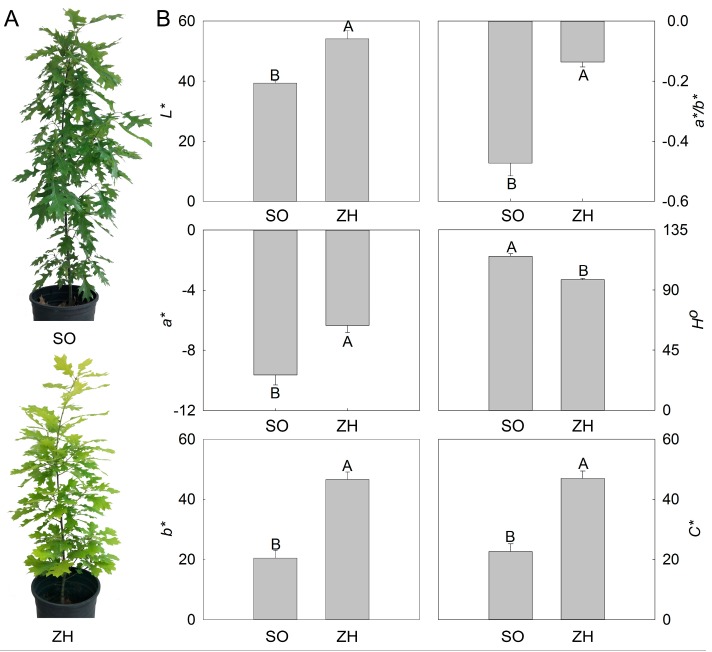
Phenotypes and color indices of ‘Shumard oak No. 23’ (‘SO’) and ‘Zhongshan Hongjincai’ (‘ZH’). (**A**) Phenotypes of ‘SO’ and ‘ZH’; (**B**) Color indices of ‘SO’ and ‘ZH’. The values represented the mean ± standard deviation (SD), and different letters indicate very significant differences according to Duncan’s multiple range test (*p* < 0.01).

To clarify the mechanism of yellow leaf coloration in *Q. shumardii*, a yellow leaf variety (‘ZH’) and a green leaf variety (‘SO’) were used as the materials (Figure 1A). The differences in leaf colors between ‘SO’ and ‘ZH’ expressed as the L^*^, a^*^, b^*^, a^*^/b^*^, *H°* and C^*^ were shown in Figure 1B. In comparison to ‘SO’, L^*^, a^*^ and b^*^ values of ‘ZH’ were all very significantly higher, and their a^*^ values were negative, which indicated that the leaf colors of ‘SO’ and ‘ZH’ were green and yellow, respectively. Moreover, the a^*^/b^*^ value of ‘SO’ was negative, while that of ‘SO’ was close to zero, and *H°* values of ‘SO’ and ‘ZH’ were 115.27° and 97.73°, respectively. These five color indices were consistent with our visual results. In addition, C^*^ value of ‘ZH’ was also very significantly higher than that of ‘SO’, showing that ‘ZH’ had better chroma.

### 2.2. SPAD Value and Pigment Content

**Figure 2:**
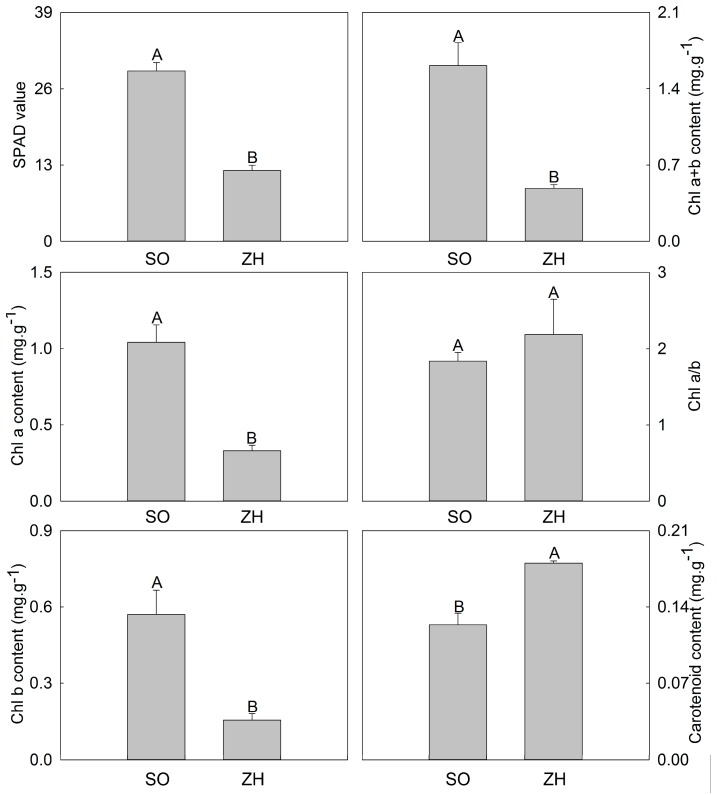
Soil plant analysis development (SPAD) value and pigment content of ‘SO’ and ‘ZH’. The values represented the mean ± SD, and different letters indicate very significant differences according to Duncan’s multiple range test (*p* < 0.01).

Subsequently, their soil plant analysis development (SPAD) value and pigment content were measured (Figure 2). SPAD value of ‘SO’ was very significantly higher than that of ‘ZH’. Moreover, Chl a, Chl b and Chl a + b contents in ‘SO’ were also very significantly higher than those in ‘ZH’, they in ‘SO’ was 314%, 368% and 331% that of ‘ZH’, respectively. Whereas, Chl a/b and carotenoid content showed the opposite tendency, the higher values were found in ‘ZH’, and the carotenoid contents between ‘SO’ and ‘ZH’ could reach highly significant level.

### 2.3. Photosynthetic Characteristics and Chlorophyll Fluorescence Parameters

**Figure 3:**
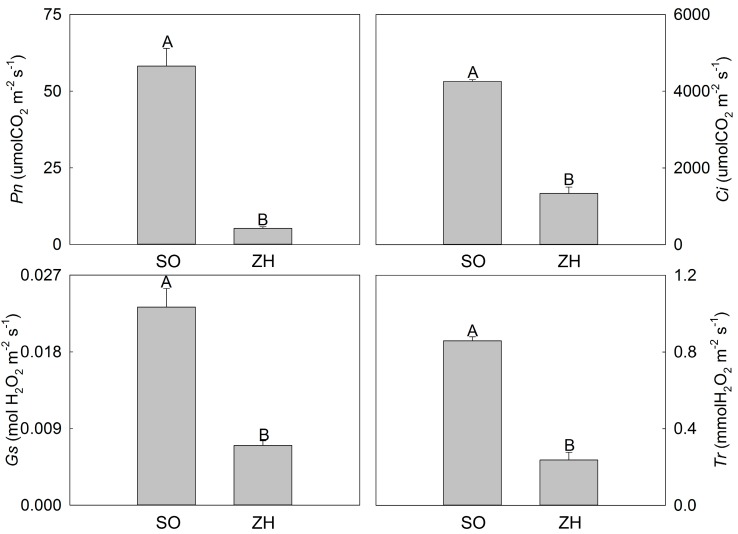
Photosynthetic characteristics of ‘SO’ and ‘ZH’. The values represented the mean ± SD, and different letters indicate very significant differences according to Duncan’s multiple range test (*p* < 0.01).

Four photosynthetic parameters were measured in this study using a portable photosynthesis system including *Pn*, intercellular CO_2_ concentration (*Ci*), stomatal conductance (*Gs*) and transpiration rate (*Tr*) (Figure 3). The *Pn* of ‘ZH’ was only 9% of ‘SO’, indicating that the photosynthetic capacity of ‘SO’ was significantly higher than that of ‘ZH’. In addition, the gas exchange parameters including *Ci*, *Gs* and *Tr* of ‘ZH’ were also significantly decreased when compared with ‘SO’, and they were only 31.44%, 30.21% and 27.54% of ‘ZH’, respectively.

**Figure 4:**
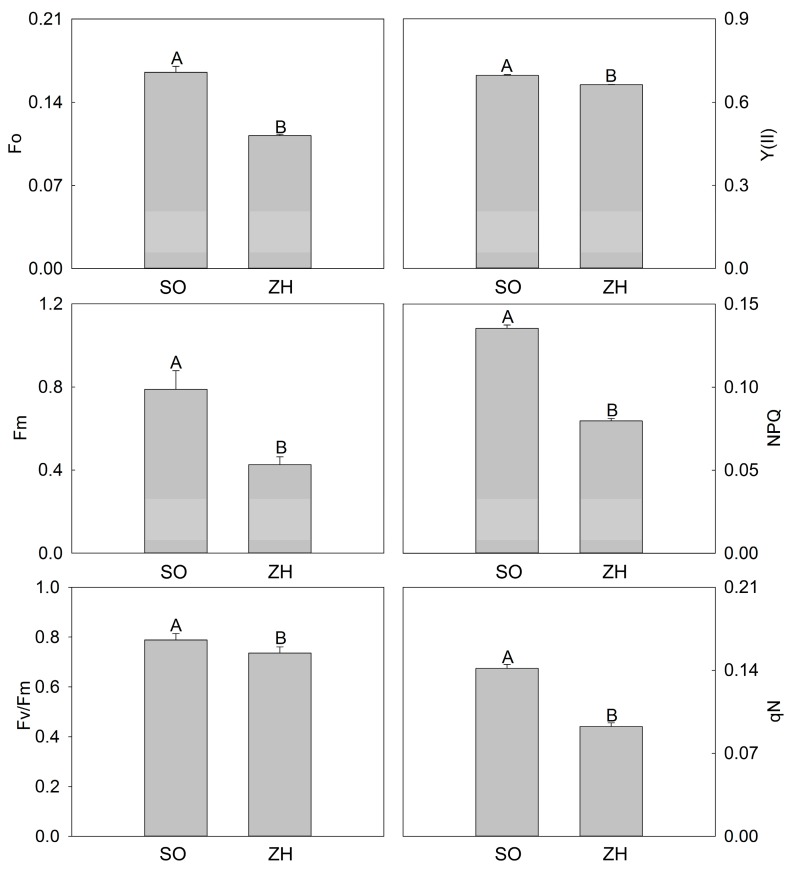
Chlorophyll fluorescence parameters of ‘SO’ and ‘ZH’. The values represented the mean ± SD, and different letters indicate very significant differences according to Duncan’s multiple range test (*p* < 0.01).

The chlorophyll fluorescence parameters of ‘SO’ and ‘ZH’ were showed in Figure 4. Similar to the photosynthetic characteristics, it was clear that the six parameters of ‘SO’ (minimum fluorescence (Fo), maximum fluorescence (Fm), nonphotochemical quenching (NPQ) and non-photochemical quenching coefficient (qN), and actual photosynthetic efficiency of photosystem II (Y(II)) and maximum quantum yield of PSII (Fv/Fm)) were all higher than those of ‘ZH’, with significant differences.

### 2.4. Anatomical Structures

**Figure 5:**
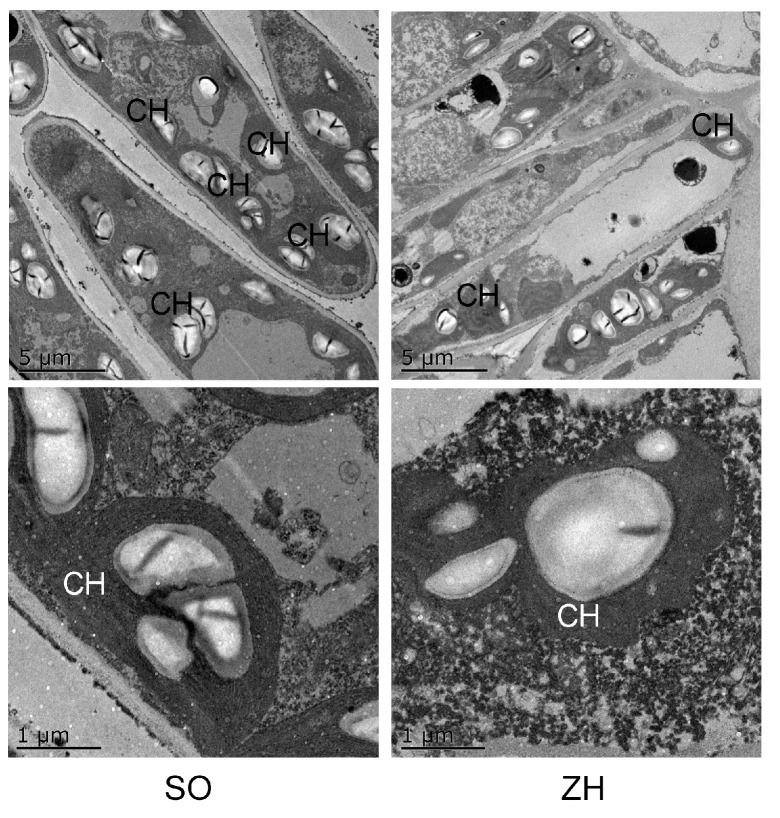
Observation of mesophyll cells of ‘SO’ and ‘ZH’. CH: chloroplast.

The anatomical structures of leaf mesophyll cells were observed by transmission electron microscopy. As shown in Figure 5, leaf mesophyll cell structures in ‘SO’ and ‘ZH’ were normal and similar to each other, but ‘ZH’ had fewer chloroplasts than ‘SO’. Moreover, the chloroplast membrane structure and the thylakoid layer structure of ‘ZH’ were destroyed, which usually occurred simultaneously with the decrease of chlorophyll [23,24]. Additionally, the decreased number of chloroplasts would help yellow coloration due to xanthophylls [25] and carotenes [26] present in the leaves.

### 2.5. Transcriptome Sequencing Results and Quality Assessment

To understand gene expression changes of yellow leaf coloration in *Q. shumardii*, six libraries from the leaves of ‘SO’ and ‘ZH’ with three biological replicates were constructed and sequenced by the high-throughput Illumina sequencing technology. An average of 51.05 M total reads with total clean bases of 7.29 Gb, Q30 percentage of 94.18% and GC percentage of 40.45% were generated from each library (Table 1). These data have been deposited in the NCBI (SRA: SRP161474). After trimming adapters, filtering out low-quality reads and de novo assembly, 154,866 unigenes with a mean length of 1059 bp and N50 of 2076 across a total of 164,100,210 bp were obtained. These data indicated that the sequencing quality was acceptable and could be used for further analysis.

### 2.6. Functional Annotation for Unigenes

All unigenes were aligned with NR (non-redundant protein sequences), NT (non-redundant nucleotide sequences), Swissprot (A manually annotated and reviewed protein sequence database), KEGG (Kyoto Encyclopedia of Genes and Genomes), KOG (Clusters of Orthologous Groups of proteins), Pfam (Protein family) and GO (Gene Ontology) databases to understand their functional information. As shown in Table S1, the alignment results showed that unigenes in the NR, NT, Swissprot, KEGG, Pfam and GO databases were 89,649 (57.89%), 75,124 (48.51%), 63,661 (41.11%), 62,055 (40.07%), 55,622 (35.92%) and 50,289 (32.47%), respectively, and the total number of unigenes obtained was 101,254 (65.38%).

**Figure 6:**
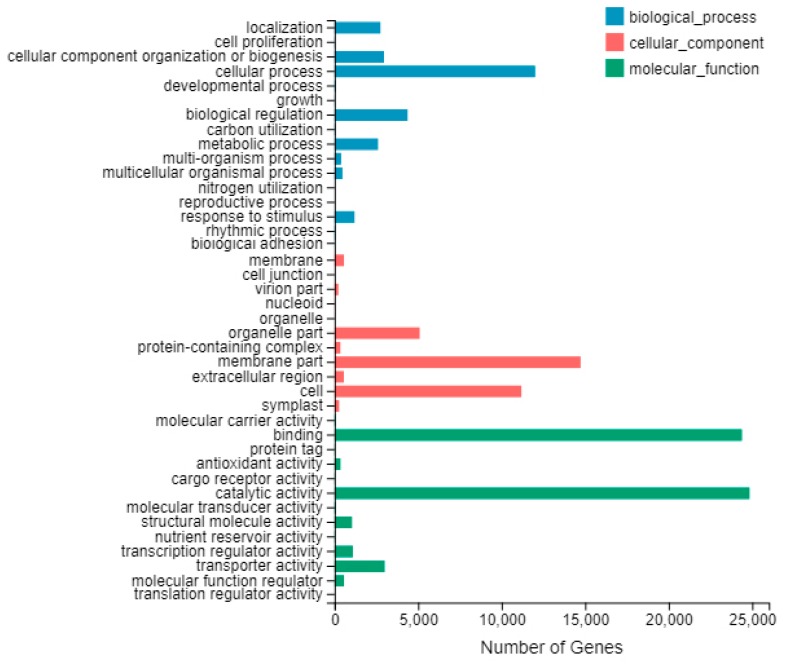
Gene ontology (GO) annotation of unigenes.

Among them, the number of unigenes annotated simultaneously by all databases was 26,907 (17.37%). In the GO functional classification, 50,289 unigenes were separately annotated into three aspects of GO: biological process, cellular component and molecular function (Figure 6). When classified according to the biological process, unigenes accounted for the highest percentage was the cellular process function, including 12,009 unigenes (23.88%). When classified according to cellular component, unigenes in membrane part and cell were 14,728 (29.29%) and 11,185 (22.24%), respectively, accounting for the highest proportion. According to the molecular function classification, unigenes accounted for the highest proportion were binding and catalytic activity functions, which were 24,387 (48.49%) and 24,843 (49.40%) unigenes.

### 2.7. DEGs Analysis and Verification

**Figure 7:**
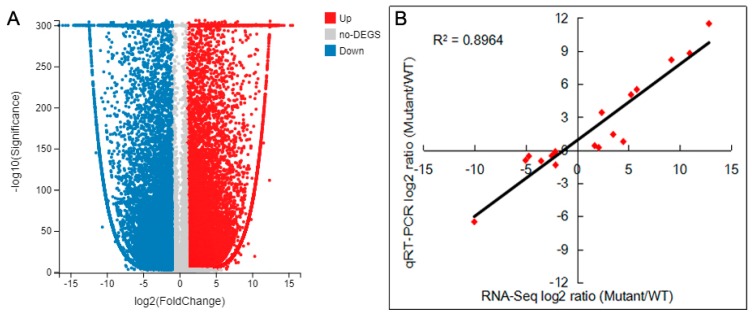
Analysis and validation of differentially expressed genes (DEGs) between ‘SO’ and ‘ZH’. (**A**) Volcano plot of DEGs. The X axis represents log2 transformed fold change; the Y axis represents -log10 false discovery rate, the red points represent up-regulated DEGs, the blue points represent down-regulated DEGs and the gray points represent non-DEGs; (**B**) correlation of gene expression results obtained from RNA-seq (X axis) and quantitative real-time polymerase chain reaction (qRT-PCR) (Y axis) analysis. Correlation assay performed for 18 DEGs with log2 ratio ≥ 1.00 or ≤ −1.00.

**Figure 8:**
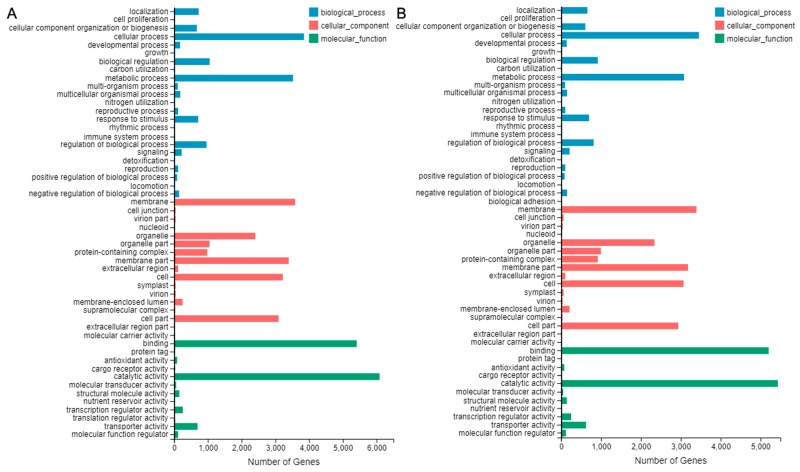
GO annotation of DEGs between ‘SO’ and ‘ZH’. (**A**) GO annotation of up-regulated DEGs; (**B**) GO annotation of down-regulated DEGs.

Subsequently, 154,866 unigenes were annotated successfully. Differentially expressed genes (DEGs) analysis was conducted between ‘SO’ and ‘ZH’. A total of 39,962 DEGs were expressed, with 20,182 up-regulated DEGs and 19,780 down-regulated DEGs (Figure 7A). Moreover, the expression levels of 18 DEGs were validated by quantitative real-time polymerase chain reaction (qRT-PCR), and we found a significant positive correlation (R^2^ = 0.896) between their results and RNA-seq data (Figure 7B), which indicated that the RNA-seq data were credible. In order to functionally classify these DEGs, they were firstly annotated to GO. The up-regulated DEGs were involved in biological processes (11,388 DEGs), cellular components (17,424 DEGs) and molecular function (11,918 DEGs), which might be closely related to the difference in leaf color between ‘SO’ and ‘ZH’, such as metabolic process (3081 DEGs), cellular process (3455 DEGs), membrane (3395 DEGs), binding (5204 DEGs) and catalytic activity (5434 DEGs) all had obvious changes (Figure 8A). Meanwhile, the down-regulated DEGs were also involved in biological processes (12,775 DEGs), cellular components (18,334 DEGs) and molecular function (12,882 DEGs), and the obvious changes contained membrane part (3395 DEGs) besides metabolic process (3523 DEGs), cellular process (3849 DEGs), membrane (3586 DEGs), binding (5409 DEGs) and catalytic activity (6087 DEGs) (Figure 8B).

**Figure 9:**
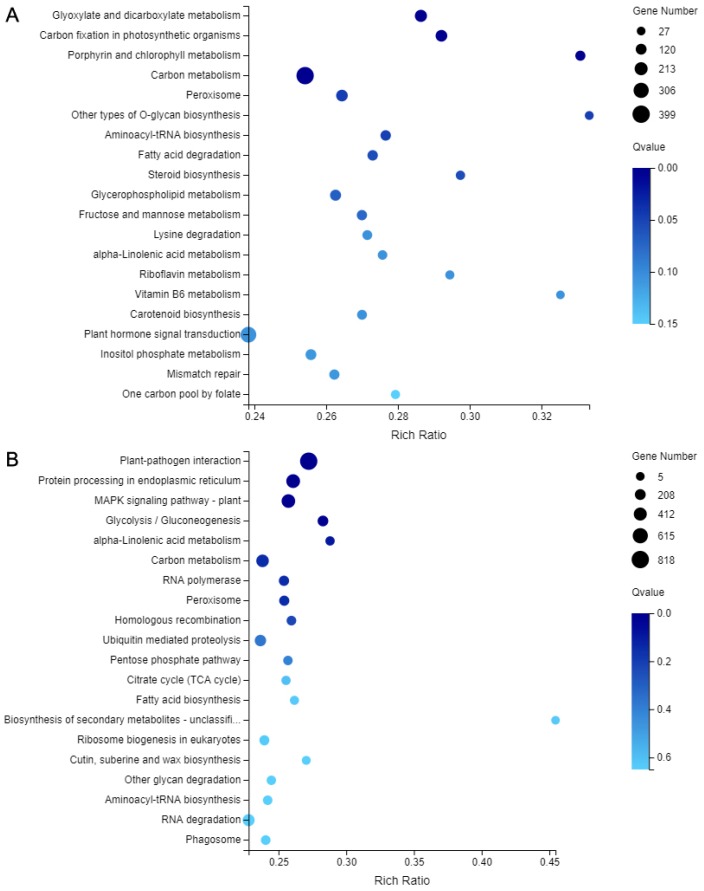
KEGG annotation of DEGs between ‘SO’ and ‘ZH’. (**A**) KEGG annotation of up-regulated DEGs; (**B**) KEGG annotation of down-regulated DEGs.

In addition, KEGG annotation was performed on all DEGs between ‘SO’ and ‘ZH’, 13,843 DEGs were obtained, corresponding to 135 pathways, and only 15 pathways met Q-value ≤ 0.05 (Table 2). Among these metabolic pathways, ‘Plant-pathogen interaction’ contained the largest number of DEGs (1419, 10.25%, ko04626), followed by ‘MAPK signaling pathway-plan’ (851 DEGs, 6.15%, ko04016), ‘Carbon metabolism’ (773 DEGs, 5.58%, ko01200), ‘Plant hormone signal transduction’ (645 DEGs, 4.66%, ko04075), ‘Glycolysis/Gluconeogenesis’ (334 DEGs, 2.41%, ko00010), ‘Peroxisome’ (306 DEGs, 2.21%, ko04146) and ‘Glycoxylate and dicarboxylate metabolism’ (292 DEGs, 2.11%, ko00630). In ‘ZH’, the down-regulated DEGs were enriched in ‘Plant-pathogen interaction’, ‘Protein processing in endoplasmic reticulum’, ‘MAPK signaling pathway–plant’ and ‘Glycolysis/Gluconeogenesis’ pathways, whereas the up-regulated DEGs were enriched in ‘Glyoxylate and dicarboxylate metabolism’, ‘Carbon fixation in photosynthetic organisms’, ‘Porphyrin and chlorophyll metabolism’ and ‘Carbon metabolism’ pathways (Figure 9).

### 2.8. Chlorophyll and Carotenoid Biosynthetic Genes Involved in Leaf Coloration

**Figure 10:**
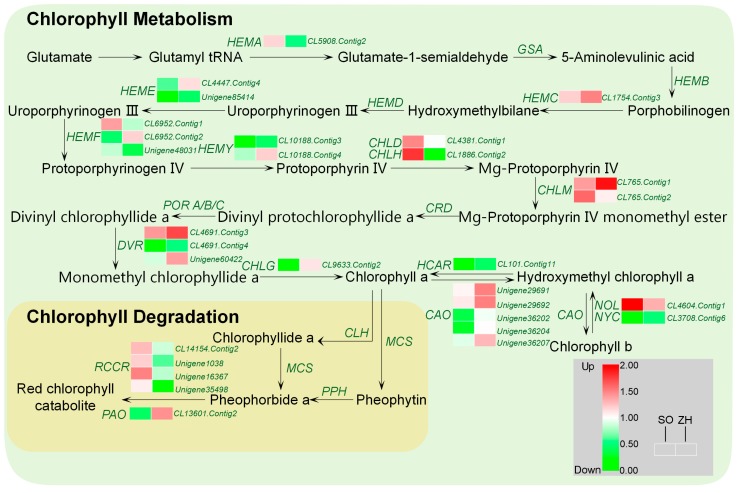
Expression profiles of DEGs involved in chlorophyll biosynthesis and degradation between ‘SO’ and ‘ZH’.

The biosynthesis and degradation of chlorophyll play a crucial role in leaf coloration. Based on the KEGG pathway assignment, 30 DEGs associated with chlorophyll metabolism were identified, including five DEGs associated with chlorophyll degradation, and their expression levels were showed by hierarchical cluster analysis (Figure 10). Among them, *CHLD* and *CHLH*, which were magnesium chelatase subunits, had significantly decreased expression levels in ‘ZH’, indicating that Mg-protoporphyrin had a lower biosynthesis rate in ‘ZH’. In addition, the expression levels of DEGs involved in the transformation of Chl a and Chl b in ‘ZH’ were mostly higher than those in ‘SO’, indicating a higher rate of conversion between Chl a and Chl b in ‘ZH’. Moreover, Chl b could only enter the degradation pathway by conversion to Chl a, and pheophorbide a oxygenase gene (*PAO*) closely related to chlorophyll degradation was significantly up-regulated in ‘ZH’. These results indicated that the rate of chlorophyll degradation in ‘ZH’ was faster than that of ‘SO’.

**Figure 11:**
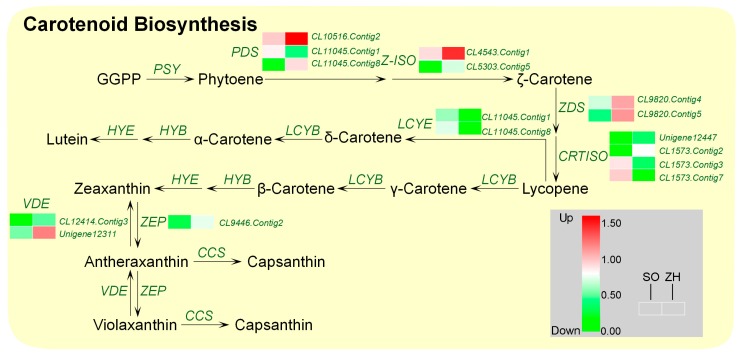
Expression profiles of DEGs involved in carotenoid biosynthesis between ‘SO’ and ‘ZH’.

Meanwhile, 16 DEGs associated with carotenoid biosynthesis were annotated in this database (Figure 11). Among them, 11 DEGs were significantly up-regulated in ‘ZH’, and five DEGs were significantly down-regulated. The up-regulated DEGs mainly contained *PDS*, ζ-carotene isomerase gene (*I-ZOS*), *ZDS*, zeaxanthin epoxidase gene (*ZEP*) and violaxanthin de-epoxidase gene (*VDE*), and the down-regulated DEGs were mainly *LCYE* and carotenoid isomerase gene (*CRTISO*). These results indicated that the expression levels of most of carotenoid biosynthetic genes in ‘ZH’ were significantly higher than those of ‘SO’.

## 3. Discussion

Leaf color is an important ornamental and commercial trait. In this study, we clearly observed the leaf color difference between ‘SO’ and ‘ZH’ of *Q. shumardii*, in which ‘SO’ was green and ‘ZH’ leaves was yellow. The color difference between ‘SO’ and ‘ZH’ was determined by an instrument in which the negative value of ‘SO’ a^*^ was lower than that of ‘ZH’, and its positive value of b^*^ was also lower. These color indices were consistent with the observed leaf color of plants [27,28]. In addition, the L^*^ value of ‘ZH’ was higher than that of ‘SO’, indicating that the yellow color of ‘ZH’ was brighter and its ornamental value was better.

Leaf coloration is the result of the combined action of different pigments. The change of pigment type and its content determines the expression of leaf color [29]. There are three main types of pigments in plant leaves: chlorophyll, carotenoids and anthocyanins. Among them, chlorophyll makes the leaves appear green, carotenoids mainly make the leaves appear yellow and anthocyanins make the leaves appear red. In this study, we mainly studied chlorophyll and carotenoids. Many chlorophyll-deficient mutants were also found in rice [30] and *Arabidopsis thaliana* [31], and the lack of chlorophyll resulted in a physiological change in leaf color. Our results showed that SPAD value, Chl a, Chl b and Chl a + b contents in ‘SO’ were all higher than those in ‘ZH’, while the carotenoid content in ‘ZH’ was significantly higher than that in ‘SO’, indicating that the combination of chlorophyll reduction and carotenoid increase might induce yellow leaf coloration of ‘ZH’. Zhang et al. [23] found that the light-harvesting antenna complex and the optical system were severely damaged in the yellow leaf mutant *pylm* of Pak-choi (*Brassica rapa*), resulting in a significant increase in Chl a/b. In this study, there was no significant difference in Chl a/b between ‘SO’ and ‘ZH’, indicating that the transformation between Chl a and Chl b in ‘SO’ and ‘ZH’ was not significantly impeded and the optical system was not severely damaged.

In general, thylakoid membranes in the chloroplasts of higher plants are regularly arranged and stacked into grana, and many leaf color mutants usually exhibit abnormal thylakoid structures [23,32]. In this study, the chloroplast structure of ‘SO’ was intact and clearly visible, the thylakoid layer structure was tight and the thylakoid membrane is regularly arranged. However, the ultrastructure of chloroplasts in ‘ZH’ changed, and the thylakoids were irregularly arranged, indicating that the chloroplasts were abnormally developed in ‘ZH’. Additionally, the significantly reduced chlorophyll content in leaves might be due to abnormal chloroplast structure in ‘ZH’. Similar results were found in other plants such as chrysanthemum mutant [33], bamboo mutant [34] and rice mutant [32].

Chlorophyll is mainly distributed in the thylakoid membrane, and the thylakoid membrane is regularly deposited into grana, which is very effective in the absorption and conversion of light energy [32]. Abnormal thylakoid structures often result in decreased photosynthetic capacity [23,32]. In this study, the photosynthetic characteristics and chlorophyll fluorescence parameters of ‘ZH’ were significantly smaller than those of ‘SO’. At the same time, this study had shown that the structural abnormalities of thylakoids in ‘ZH’, which usually occurred simultaneously with the decrease of chlorophyll [23,24]. Therefore, the lower photosynthetic characteristics and chlorophyll fluorescence parameters of ‘ZH’ in this study might be caused by abnormal changes in chloroplast structure and decreased chlorophyll content.

Comparative transcriptomic analysis could reveal gene expression changes [35]. In this study, Illumina HiSeq 4000 high-throughput sequencing technology was used to perform transcriptome sequencing of ‘SO’ and ‘ZH’. A total of more than 50 M of raw readings were obtained for each treatment group, the totals of clean bases obtained in each group were above 7 Gb, the percentage of filtered Q30 was also greater than 93% and the percentage of G and C bases in reads was about 40%. Wang et al. [36] performed transcriptomic analysis of loquat with a Q30 base of 92.5%; Li et al. [37] believed that the percentage of G and C bases in reads should be between 35% and 65%. Compared with this, the sequencing quality was good.

Based on the transcriptome data, a total of 39,962 DEGs were identified between ‘SO’ and ‘ZH’. In addition, it was worth noting that our data analysis focused on some of the DEGs associated with chlorophyll biosynthesis and degradation and carotenoid biosynthesis might be involved in leaf coloration of *Q. shumardii*. In *Arabidopsis thaliana*, a total of 15 enzymes were involved in chlorophyll biosynthesis, and these 15 enzymes were encoded by 27 genes [11]. In this biosynthetic process, any step would block the biosynthesis of chlorophyll and affect the chlorophyll content [2]. Zhang et al. [38] found in the study of rice leaf color mutants chl1 and chl9 that the chlorophyll biosynthesis resistance site of this mutant occurred at the site of protoporphyrinogen IX to magnesium protoporphyrin; Li et al. [39] studied the differences between white and green leaves of pineapple and found that the white leaves were blocked during the biosynthesis of porphobilinogen (PBG) to uroporphyrinogen III, resulting in changes in leaf color. All these above studies had shown that as long as one site of transformation and biosynthesis was blocked during the whole process of chlorophyll biosynthesis, the content of the products before the blocked site would increase, and the content of all products after the blocked site would decrease, which would directly lead to impaired chlorophyll biosynthesis, resulting in decreased leaf greenness. In this study, based on the distribution of the KEGG pathway, 30 DEGs associated with chlorophyll metabolism were identified. It was apparent that *CHLD* and *CHLH* that regulated the transformation of protoporphyrin IX to Mg-protoporphyrin IX were significantly down-regulated, suggesting that the chlorophyll biosynthesis resistance of Mutant might be located in this step. Among them, the expression of *CHLH* was mainly regulated by illumination affecting the expression of photoreceptor early light inducible protein (ELIP), resulting in changes in chlorophyll biosynthesis ability. For example, the inhibition of *CHLH* expression in *Nicotiana tabacum* lead to a decrease in chlorophyll biosynthesis rate [40]. Moreover, when the leaf color of the plants changed, the transcription levels of *CHLH*, *HEMA* and *HEMD* decreased as the chlorophyll content decreased [8,12]. In addition, *HEMA* encoded glutamyl-tRNA reductase, which was the initial enzymatic step in the biosynthesis of chlorophyll [41]. In this study, the expression level of *HEMA* in ‘ZH’ was decreased, which was consistent with previous studies [41]. Therefore, it could be considered that the down-regulation of *HEMA*, *CHLD* and *CHLH* might be the key to the decrease in the efficiency of ‘ZH’ chlorophyll biosynthesis. Moreover, carotenoids are a family of pigments, ranging in color to yellow to red, which are involved in light harvesting and are indispensable for photoprotection under conditions of excess light [15,42]. Therefore, the up-regulated expression levels of carotenoid biosynthesis-related genes might lead to yellowing of the leaves [43]. In this study, 11 DEGs related to carotenoid biosynthesis were identified, in which *Z-ISO*, *ZDS*, *VDE* and *ZEP* expression were significantly up-regulated in ‘ZH’. This enhanced carotenoid biosynthesis gene expression and increased carotenoid content in ‘ZH’ indicated that carotenoids contributed to yellow leaf coloration in ‘ZH’. These results could provide valuable information for understanding the mechanism of yellow leaf coloration in *Q. shumardii*.

## 4. Materials and Methods

### 4.1. Plant Materials

A yellow leaf variety of *Q. shumardii* named ‘Zhongshan Hongjincai’, and a green leaf variety named ‘Shumard oak No. 23’ were used as the materials in this study. They were obtained by sowing the seeds of *Q. shumardii* introduced from the United States. These plants were asexually propagated by grafting and preserved in Jiangsu Academy of Forestry, Jiangsu Province, China (31°51′29′′ N, 118°45′59′′ E). These potted plants were collected in June, 2019. First, the measurements of leaf color indices, photosynthetic characteristics and chlorophyll fluorescence parameters were performed, and then the samples were stored at −80 °C.

### 4.2. Color Indices Measurement

Color indices of leaf were measured with a hand-held RM200QC spectrocolourimeter (X-Rite, Grand Rapids, MI, USA) using six color parameters including L^*^, a^*^, b^*^ values. The hue angle (*H°* = arctangent (b^*^/a^*^)) and chroma (C^*^= (a^*2^ + b^*2^)^1/2^) were calculated according to the methods reported previously [44]. In the uniform color space, L^*^ represents the lightness, a^*^ represents the ratio of red/magenta to green and b^*^ represents the ratio of yellow to blue [45].

### 4.3. SPAD Value and Pigment Content Measurement

SPAD value was determined using a SPAD-502 chlorophyll meter (Konica Minolta Sensing, Tokyo, Japan). Chl a, Chl b, Chl a + b, Chl a/b and carotenoid contents were assayed according to Zou [46]. Moreover, the absorbance was read at 665 nm, 649 nm and 470 nm on a spectrophotometer UV BlueStar A (Beijing LabTech Instruments Co., Ltd., Beijing, China).

### 4.4. Photosynthetic Characteristics and Chlorophyll Fluorescence Parameters Measurement

Photosynthetic characteristics were measured using a LI-6400 portable photosynthesis system (Li-cor, Lincoln, NE, USA) from 7:00 to 9:00 am on a cloudless day. The standard leaf chamber was 2 cm × 3 cm in size. Additionally, in this system, *Pn*, *Ci*, *Gs* and *Tr* were recorded. Moreover, a chlorophyll fluorescence spectrometer (Heinz Walz GmbH 91090, Effeltrich, Germany) was used to measure the chlorophyll fluorescence parameters. Furthermore, this system recorded Fo, Fm, NPQ and qN, and Y(II) together with Fv/Fm were calculated [47,48].

### 4.5. Anatomy Observation

A Tecnai 12 transmission electron microscope (Philips Electron Optics, Eindhoven, Netherlands) was used to observe the anatomical details of leaves. The fixed leaves were washed three times with 0.1 mol/dm^3^ phosphate buffer for 15 min, and post-fixed with 1% osmium tetroxide for 4 h at room temperature (25 °C). After washing 3 times with 0.1 mol/dm^3^ phosphate buffer for 15 min each, the leaves were dehydrated using 50%, 70%, 85%, 95% and 100% gradient ethanol for 15 min each. Moreover, they were treated with 100% acetone solution (15 min) and acetone solution containing anhydrous sodium sulfate (15 min), infiltrated in spurr resin and then hardened at 70 °C for 24 h. Sections (70 nm thickness) were cut with a diamond knife using a Leica EM UC6 ultramicrotome (Leica Co., Wetzlar, Germany) and stained with 1% uranyl acetate in 70% methanol, and 1% lead citrate before examination. Finally, the samples were observed on metal plates, the accelerating voltage was 50–100kV, the magnification of the whole cells was 2850 times, and that of chloroplasts was 13,500 times.

### 4.6. RNA-seq and Data Analysis

Total RNAs extracted from the leaves of ‘SO’ and ‘ZH’ using a MiniBEST Plant RNA Extraction Kit (TaKaRa, Tokyo, Japan) was used for transcriptome sequencing. After the determination of total RNA concentration and purity, six libraries (‘SO’ and ‘ZH’, three replicates) were prepared and sequenced by Beijing Genomic Institute (Shenzhen, China) using an Illumina HiSeq™ 4000 platform (Illumina Inc., San Diego, CA, USA). After raw reads filtering, transcriptome de novo assembly was performed using short reads assembling program Trinity [49]. In addition, the resulting sequences of Trinity were called unigenes, and various bioinformatics databases were used for their annotation.

The unigene expression was calculated and normalized to Reads Per kilo bases per Million reads (RPKM) [50]. GO functional analysis and KEGG pathway analysis were performed on DEGs based on fold change ≥ 2.0 and adjusted *p*-value ≤ 0.05.

### 4.7. Gene Expression Analysis

qRT-PCR was used to detect gene expression levels with a BIO-RAD CFX Connect^TM^ Optics Module (Bio-Rad, Des Plaines, IL, USA), and their values were calculated referring to the 2^−^^△△Ct^ comparative threshold cycle (Ct) method [51]. The cDNA was synthesized from RNA using PrimeScript^®^ RT reagent Kit With gDNA Eraser (TaKaRa, Tokyo, Japan). qRT-PCR was performed using the SYBR^®^ Premix Ex Taq^TM^ (Perfect Real Time) (TaKaRa, Tokyo, Japan) and contained 12.5 mm^3^ 2 × SYBR Premix Ex Taq^TM^, 2 mm^3^ cDNA solution, 2 mm^3^ mix solution of target gene primers and 8.5 mm^3^ ddH_2_O in a final volume of 25 mm^3^. The amplification was carried out under the following conditions: 95 °C for 30 s, 40 cycles at 95 °C for 5 s, 52 °C for 30 s and 72 °C for 30 s. All used primers were listed in Table S2.

### 4.8. Statistical Analysis

All experiments described here were repeated three times arranged in a completely randomized design. Primers were designed using a Primer 5.0 program (Premier Biosoft, Palo Alto, CA, USA). All data were means of three replicates with standard deviations. The results were analyzed for variance using the SAS/STAT statistical analysis package (version 6.12, SAS Institute, Cary, NC, USA).

## Figures and Tables

**Table 1:** Output statistics of RNA-seq of ‘SO’ and ‘ZH’.

Sample	Total Raw Reads (M)	Total Clean Bases (Gb)	GC (%)	Q30 (%)	Adapter (%)	Low Quality (%)
SO-1	52.42	7.46	40.40	93.57	0.63	4.49
SO-2	50.78	7.28	40.40	95.23	0.56	3.89
SO-3	50.78	7.24	40.47	93.66	0.46	4.42
ZH-1	50.78	7.25	40.45	93.73	0.52	4.24
ZH-2	50.78	7.29	40.44	95.25	0.57	3.73
ZH-3	50.78	7.24	40.55	93.61	0.59	4.32
Average	51.05	7.29	40.45	94.18	0.56	4.18

**Table 2:** Kyoto encyclopedia of genes and genomes (KEGG) pathways with pathway identity information.

No.	Pathway Name	DEGs Num.	Pathway ID
1	Carbon metabolism	773 (5.58%)	ko01200
2	Plant-pathogen interaction	1419 (10.25%)	ko04626
3	Porphyrin and chlorophyll metabolism	152 (1.10%)	ko00860
4	Peroxisome	306 (2.21%)	ko04146
5	alpha-Linolenic acid metabolism	137 (0.99%)	ko00592
6	Carbon fixation in photosynthetic organisms	274 (1.98%)	ko00710
7	Mitogen-activated protein kinase (MAPK) signaling pathway - plan	851 (6.15%)	ko04016
8	Aminoacyl-tRNA biosynthesis	195 (1.41%)	ko00970
9	Glycolysis / Gluconeogenesis	334 (2.41%)	ko00010
10	Homologous recombination	197 (1.42%)	ko03440
11	Carotenoid biosynthesis	153 (1.11%)	ko00906
12	Plant hormone signal transduction	645 (4.66%)	ko04075
13	Steroid biosynthesis	103 (0.74%)	ko00100
14	Fatty acid degradation	189 (1.37%)	ko00071
15	Glyoxylate and dicarboxylate metabolism	292 (2.11%)	ko00630

## Supplementary Materials

The following are available online at https://www.mdpi.com/2223-7747/9/2/169/s1, Table S1: Statistics of annotation results, Table S2: Primers used for quantitative real-time polymerase chain reaction (qRT-PCR).
