# Supplementary material for: Physiological and Anatomical Differences and Differentially Expressed Genes Reveal Yellow Leaf Coloration in Shumard Oak
Source: Plants (Basel). 2020 Feb 1;9(2):169. doi: 10.3390/plants9020169 (PMC7076436; doi:10.3390/plants9020169)
Supplement: Supplementary file 1 [file plants-09-00169-s001.zip › plants-705603-supplementary/Non-published Material/Table S1.doc]

**Table S1.** Statistics of annotation results.

| Values | Total | NR | NT | Swissprot | KEGG | Pfam | GO | Intersection | Overall |
| --- | --- | --- | --- | --- | --- | --- | --- | --- | --- |
| Number | 154,866 | 89,649 | 75,124 | 63,661 | 62,055 | 55,622 | 50,289 | 26,907 | 101,254 |
| Percentage | 100% | 57.89% | 48.51% | 41.11% | 40.07% | 35.92% | 32.47% | 17.37% | 65.38% |

Intersection: The number of genes annotated simultaneously by all databases. Overall: The number of genes annotated on any database.
