# Supplementary material for: Physiological and Anatomical Differences and Differentially Expressed Genes Reveal Yellow Leaf Coloration in Shumard Oak
Source: Plants (Basel). 2020 Feb 1;9(2):169. doi: 10.3390/plants9020169 (PMC7076436; doi:10.3390/plants9020169)
Supplement: Supplementary file 1 [file plants-09-00169-s001.zip › plants-705603-supplementary/Non-published Material/Table S2.doc]

**Table S2. Primers used for quantitative real-time polymerase chain reaction (qRT-PCR).**

| Gene ID | Gene name | Forward primer (5' - 3') | Reverse primer (5' - 3') |
| --- | --- | --- | --- |
| Unigene9852_All | actin | TCGAAAGCCTACTCAAACA | AGAGCAATCCTTCCACATC |
| CL4381.Contig2_All | magnesium chelatase subunit D gene | CTTCCTCGTTCAATCATCA | CCTCTTGTTCGTCCTCCTC |
| CL6090.Contig1_All | Frataxin gene | AGCCAATTCCACAATACATC | TCAACACCTCATTCCCATA |
| Unigene27867_All | (S)-hydroxynitrile lyase gene | TCCAGCAGAGGAAAGAGTG | TCAGGACCAGGCATAAGG |
| CL8613.Contig2_All | pheophorbide a oxygenase gene | AAAAGCATCCTTGGAGTG | AAAGTTGAGTCTTGGGGTAT |
| Unigene17105_All | pheophorbide a oxygenase gene | CATACAAAGGTCTCAATGCA | AGCCGTGGTAATGGAAGT |
| CL4978.Contig1_All | protochlorophyllide reductase gene | CACAGCTAAGGAGCCATC | GTCATCAAGCAGTAACCGA |
| Unigene301_All | divinyl chlorophyllide a 8-vinyl-reductase gene | TGCCTATACTAGAGGAGAAATC | GACCAGCAAATGTGAGAAG |
| CL531.Contig5_All | protoporphyrinogen/coproporphyrinogen III oxidase gene | TTGTGGCATGAAGAACAGA | AGCAAAAGTGGGGAAAAC |
| CL5908.Contig2_All | glutamyl-tRNA reductase gene | CGTGAAGAGTTGAAGGGTG | ATAGTGGGGTTTCCGATG |
| CL4604.Contig1_All | chlorophyll(ide) b reductase gene | GAGGTCGCTGGTTTGATG | GGAAATGTGCTGCTAGTGC |
| CL7710.Contig1_All | protein phosphatase inhibitor 2 gene | TATCCGACTTTCCGCTCTT | CATCTTACACGCCCCTTC |
| CL10516.Contig2_All | 15-cis-phytoene desaturase gene | CTCAAACCCCAAGCATCA | CGAGGAGGAGGAAAGGAA |
| CL10516.Contig3_All | 15-cis-phytoene desaturase gene | CCACAACAATTAGGGGACA | ACGGCAACTACGACACCA |
| CL9820.Contig3_All | zeta-carotene desaturase gene | ACGGTTGGGTCACAGAGT | GAGATGTTAGTGCTAAGTCGG |
| CL9820.Contig5_All | zeta-carotene desaturase gene | AGATACAATGGTTGGGTCA | GAAGGGTAACAGAAGCAGAT |
| CL3436.Contig3_All | lycopene epsilon-cyclase gene | GTAAAAGGTGGTGGGTCC | TGAGTCCTAACTTGGCTGA |
| CL5627.Contig14_All | zeaxanthin epoxidase gene | TTCGAGTTAAGGTATTGAGGAC | TCAGCAAGTTACGGCAGA |
| CL5862.Contig2_All | 9-cis-epoxycarotenoid dioxygenase gene | GGTGCTTTATCTGCTGCTC | GTTTCCCATCAAAATCGTAG |
